# Mirtazapine decreased cocaine-induced *c-fos* expression and dopamine release in rats

**DOI:** 10.3389/fpsyt.2024.1428730

**Published:** 2024-08-12

**Authors:** Susana Barbosa-Méndez, Alberto Salazar-Juárez

**Affiliations:** Subdirección de Investigaciones Clínicas, Laboratorio de Neurofarmacología Conductual, Microcirugía y Terapéutica Experimental, Instituto Nacional de Psiquiatría, Ciudad de México, Mexico

**Keywords:** multi-target drugs, cocaine, locomotor activity, locomotor sensitization, mirtazapine, pharmacotherapy

## Abstract

**Introduction:**

Chronic cocaine exposure induces an increase in dopamine release and an increase in the expression of the Fos protein in the rat striatum. It has been suggested that both are necessary for the expression of cocaine-induced alterations in behavior and neural circuitry. Mirtazapine dosing attenuated the cocaine-induced psychomotor and reinforcer effects.

**Methods:**

The study evaluates the effect of chronic dosing of mirtazapine on cocaine-induced extracellular dopamine levels and Fos protein expression in rats. Male Wistar rats received cocaine (10 mg/Kg; i.p.) during the induction and expression of locomotor sensitization. The mirtazapine (30 mg/Kg; MIR), was administered 30 minutes before cocaine during the cocaine withdrawal. After each treatment, the locomotor activity was recorded for 30 minutes. Animals were sacrificed after treatment administration. Dopamine levels were determined by high-performance liquid chromatographic (HPLC) in the ventral striatum, the prefrontal cortex (PFC), and the ventral tegmental area (VTA) in animals treated with mirtazapine and cocaine. The quantification of c-fos immunoreactive cells was carried out by stereology analysis.

**Results:**

Mirtazapine generated a decrease in cocaine-induced locomotor activity. In addition, mirtazapine decreased the amount of cocaine-induced dopamine and the number of cells immunoreactive to the Fos protein in the striatum, PFC, and VTA.

**Discussion:**

These data suggest that mirtazapine could prevent the consolidation of changes in behavior and the cocaine-induced reorganization of neuronal circuits. It would explain the mirtazapine-induced effects on cocaine behavioral sensitization. Thus, these data together could support its possible use for the treatment of patients with cocaine use disorder.

## 1 Introduction

Cocaine use disorder (CUD) is a significant health problem worldwide (1). There are currently no FDA-approved drugs for treatment (2).

Various studies in rodents have described that cocaine produces its psychomotor and reinforcing effects primarily by blocking the dopamine transporter (DAT) (3); this action increases extracellular dopamine levels in the striatum (4, 5), which leads to the activation of dopamine D_1_ and D_2_ receptors in the striatum, ventral-tegmental area (VTA) and prefrontal cortex (PFC) (6). However, several studies support that dopamine D_1_ receptors are necessary to mediate the cocaine-induced behavioral, cellular, and molecular effects (7–9).

In this sense, pioneering studies have documented that repeated cocaine exposure induces an increase in the expression of the Fos protein in the rat striatum (10–12). The *c-fos* gene belongs to the early expression genes (IEG) (13–15). IEGs encode transcription factors (Fos, FosB) or effector proteins (Arc, Homer) involved in various brain functions. Still, it has been suggested that they are the link that ensures the continuity between rapid synaptic changes and the adaptations required for neural plasticity (16–18). In the case of the *c-fos* gene and the Fos protein, they have been associated with cell proliferation, differentiation, transformation, and death (15, 19).

Several studies have reported that the expression of the c-fos gene is related to the persistent changes induced by cocaine (20, 21). These studies suggest that the *c-Fos* gene is an intracellular regulator of cocaine-induced alterations in gene expression, reorganization of neural circuitry, and expression of behavioral sensitization (22, 23).

In addition, animal studies have shown that various external stimuli that increase neuronal activity induce *c-fos* expression (15, 24). These studies show that in the basal condition, the constitutive levels of c-fos are low. Still, in response to an external stimulus, the c-fos levels increase rapidly and return to their basal level in a matter of hours. This property has given the expression of the c-fos gene to be considered an important experimental tool as a marker of neuronal activity (15, 25).

On the other hand, we have recently reported a series of pre-clinical and clinical studies (26, 27), which evaluated the efficacy of mirtazapine (MSD REMERON, Schering-Plough-Organon, USA), an effective noradrenergic and a specific serotoninergic antidepressant with pronounced early anxiolytic effects in patients with moderate-to-severe depression (28, 29) to attenuate the reinforcing and psychomotor effects of cocaine, nicotine, and heroin, in rodents and humans (27, 30–34).

These studies showed that dosing of mirtazapine in rodents decreases cocaine- and nicotine-induced locomotor activity (27, 30, 32), attenuates the induction and expression of methamphetamine-, morphine-, nicotine-, and cocaine-induced locomotor sensitization (27, 30, 35, 36) and place preference (CPP) (35 37–37), and reduces self-administration of methamphetamine, cocaine, nicotine, and heroin (31, 33, 34, 38, 39). 30 mg/Kg of mirtazapine decreases depression- and anxiety-like behaviors during cocaine withdrawal (40). In humans, mirtazapine reduces alcohol, cocaine, and methamphetamine abuse (26, 41–43). It also improves symptoms of depression, anxiety, and insomnia during benzodiazepine, methamphetamine, alcohol, and cocaine withdrawal (26, 41, 43–45).

Thus, given that chronic exposure to cocaine induces an increase in extracellular dopamine levels through the blockade of DATs (4) and it produces the expression of early expression genes (c-fos) via activation mainly of dopamine D_1_ receptors (9) and since both, the increase in dopamine levels and the expression of the Fos protein are necessary for the expression of cocaine-induced alterations in behavior and neural circuitry (22, 23) and since the expression of c-fos is considered a marker of neuronal activity (15), and since mirtazapine is effective in attenuating the cocaine-induced psychomotor and reinforcers effects (34), then it would be important to evaluate the effect of chronic dosing of mirtazapine on the cocaine-induced extracellular dopamine levels and Fos protein expression in rats.

## 2 Materials and methods

### 2.1 Animals

We used male Wistar rats weighing 250–280 g at the beginning of the study. They were housed in groups of four in standard plastic rodent cages (57 cm x 35 cm x 20 cm) in a colony room maintained at constant temperature (21 ± 2°C) and humidity (40–50%) on a 12:12-h light/dark cycle (lights on at 7:00 a.m.) for an acclimation period of 3 days. During this period, the animals had continuous access to rodent chow pellets and water, except during the experimental sessions. All experiments took place during the light phase of the light/dark cycle (between 9:00 a.m. and 7:00 p.m.). The Institutional Animal Care- and Bioethics Committee approved the procedures (CEI/C/IC092020/2006) in strict compliance with the Guide for the Care and Use of Laboratory Animals published by the National Institutes of Health (NIH).

### 2.2 Drugs

The Mexican government kindly donated cocaine hydrochloride (COC) under strict regulatory controls. All drugs used in experimental animals were kept under official surveillance (COFEPRIS- LC-0004-2003). Mirtazapine (MIR; Remeron, Schering-Plough-Organon-SANFER), was purchased after obtaining the required regulatory permission, as per official guidelines (COFEPRIS-2016, Mexico).

The MIR and COC were dissolved and diluted in a sterile saline solution (0.9% NaCl, Sigma Aldrich). The solutions were freshly prepared before their intraperitoneal (i.p.) administration to the animals. The pH was adjusted to seven. During the experiments, the solutions were maintained at 4°C.

To determine if MIR can prevent the effects of cocaine, these were administered 30 minutes before cocaine or saline administration. The volume injected into the animals depended on their body weight (BW) in kilograms (BW (kg)/1ml).

#### 2.2.1 Dose selection

The determination of the optimal dose of cocaine was based on previous studies. They reported that 10 mg/kg of cocaine induces a robust increase in locomotor activity and behavioral sensitization (32). This dose of cocaine does not cause seizures or lethality (32).

The optimal mirtazapine dose (30 mg/kg) was that of previous studies. They showed that ≥30 mg/kg mirtazapine does not affect spontaneous locomotor activity or produce sedation in rats, nor does it induce weight gain (46, 47). Preclinical and clinical trials have reported that 30 mg/kg of mirtazapine decreases cocaine-induced locomotor activity (27) and place preference (48).

### 2.3 Behavioral sensitization procedure

#### 2.3.1 Apparatus

For each animal, we assessed locomotor activity in transparent Plexiglass activity chambers (50 x 50 x 30 cm) connected to a PC. Each chamber had a 16x16 photocell beam array located 3 cm from the floor surface to scan locomotor activity (OMNIALVA, Instruments, Mexico). Photobeam interruptions were automatically quantified with OABiomed software (1.1) and analyzed afterward. We defined locomotor activity as the continuous horizontal locomotor activity performed by a rat, which generates the simultaneous interruption of several photo beams (OMNIALVA, Mexico).

#### 2.3.2 Procedure

We estimated spontaneous locomotor activity with a standard protocol (27). The animals were habituated to the activity chambers in three 30-minute sessions and were randomly assigned to different experimental procedures.

### 2.4 Dopamine and serotonin determination

Dopamine was determined with an HPLC standard protocol (49).

#### 2.4.1 Tissue preparation

The animals were decapitated after treatment administration. The brain was rapidly removed, and the striatum containing the NAcc shell, prefrontal cortex (PFC), and VTA were dissected using blunt-tip curved microdissecting forceps on ice. The dissected tissue was placed into cryovials and then submerged in isopentane for snap freezing. The cryovials were placed on dry ice and then stored at -80°C. We analyzed the samples using high-pressure liquid chromatography (HPLC) and electrochemical detection.

#### 2.4.2 Tissue homogenate preparation

Immediately before HPLC, the tissue was removed from the freezer, weighed, and homogenized. We performed homogenization using 400 µl of a solution containing 5% ascorbic acid, 200 mM sodium phosphate, 2.5 mM L-cysteine, 2.5 mM EDTA, and a Tekmar homogenizer for 20 seconds. Proteins were precipitated by adding 100 µl of 0.4 M perchloric acid followed by incubation at 20°C for 20 min. We collected supernatants containing DA after centrifugation at 12,000 rpm for 10 min (4°C). The samples were placed on ice and processed as soon as possible to prevent degradation. A portion of the supernatant (50 µl) was extracted and analyzed by HPLC to determine the DA concentration.

#### 2.4.3 HPLC

Dopamine, serotonin and their metabolites (DOPAC-3,4-dihydroxyphenylacetic acid, HVA-Homo vanillic acid and HIIAA-5-hydroxyindoleacetic acid) concentrations were determined by reverse-phase HPLC (RP-HPLC) in a system equipped with two PU-2089 pumps (Jasco, Inc), a degasser (Jasco, Inc), an As-2057 autosampler (Jasco, Inc), and an XL-3120fp fluorescence detector (Jasco, Inc). Millenium 32 software (Waters™) controlled the instruments. A Jupiter C18 column (300 Å, 5 μ, 4.6 × 250 mm, Phenomenex^®^) was used at 30°C. We performed column equilibration with 0.1% trifluoroacetic acid as mobile phase A (MPA). As mobile phase B (MPB), we used a linear gradient from 0.1% trifluoroacetic acid in acetonitrile up to 20% MPB within 10 minutes (from min 5 to min 15). Then, 20% MPB was maintained up to min 20, with a flow rate of 0.8 ml/min.

The fluorescence detector was set at 280/315 nm excitation/emission, 32 attenuations, with the gain at 100 and a response time of 20 s. The sample injection volume was 50 μl.

### 2.5 Histology and immunohistochemistry

Rats were anesthetized with an overdose of pentobarbital sodium (Sedal-Vet, 65 mg/ml) and were perfused transcardially with ∼250 ml of 0.9% saline followed by 250 ml of 4% paraformaldehyde, 1.4% lysine, and 0.2% sodium m-periodate (PLP) fixative in PBS (0.1 M, pH 7.2). Brains were removed; postfixed for 1 h in PLP; and cryoprotected in 10, 20, and 30% sucrose for 24 h. Brains were frozen and cut at -18°C in horizontal sections of 40 μm to obtain complete slides of the brain. We serially collected sections in four sets.

One set was stained with cresyl violet acetate (Nissl), and a second set was processed for immunohistochemistry for c-fos. The sections were incubated for 72 h at 4°C in the primary antibody (rabbit anti-Fos; CAT SC-52, Santa Cruz) diluted 1: 2,500 in PBS, 1% goat serum, and 0.3% Triton X-100 (PBSGT). Tissue was then incubated in biotinylated secondary antibody (goat anti-rabbit; CAT PK-61–01, Vector Laboratories) diluted 1:200 in PBSGT for 2 h at room temperature, followed by incubation in an avidin-biotin complex (0.9% avidin and 0.9% biotin solutions; CAT PK-61–01, Vector Laboratories) in PBSGT for 2 h at room temperature. The tissue was then reacted in diaminobenzidine (0.5 mg/ml, in Trizma buffer 7.2) with hydrogen peroxide (35 μl, 30% H2O2). Between each step, the tissue was rinsed three times for 10 minutes in PBS. The tissue was mounted on gelatin-coated slides, and the label was intensified with osmium tetroxide 0.1% for 30 s (Baker Analyzed), dehydrated with alcohol, cleared with xylene, and placed under a coverslip with Permount.

#### 2.5.1 Cell count

To quantify c-fos expression in the mesocorticolimbic dopaminergic system, four representative sections were selected under the stereotaxic atlas from (50). A first ventral section (interaural 11.70 mm) was selected to quantify c-fos-IR in the infralimbic cortex (IL). A second section (interaural 10.00 mm) contained the nucleus accumbens shell (AcbSh) and core (AcbC). A third section (interaural 3.70 mm) was used to sample the ventral tegmental area (VTA).

Images of the corresponding Nissl sections were obtained to identify and define the area for each nucleus. Images of the immunohistochemical preparation were obtained using a computerized image analysis system (Leica, Qwin Image Analyzer Imaging Research) attached to a Leica DM500 light microscope (BHT). For large nuclei, such as the AcbSh, AcbC, and VTA, the number of IR-c-fos cells was counted from photographs of 6 brain slices per rat from the 6–10 brain slices that comprise its rostro caudal extent. For smaller nuclei, such as the IL, it was sufficient to select 4 slices per rat. A grid of 20 × 20, 400-μm^2^ squares was superimposed onto the center of each nucleus, and only c-fos-IR cells on the left side of each section were manually counted at a 10× magnification. To minimize the number of false positives, background optical density (OD) was established in a nearby region lacking c-fos-IR. When the observer marked a c-fos-IR cell, the program showed its OD, and stained cells that reached or surpassed three times the background OD were positive and were counted. In contrast, cells under this staining threshold were not considered. The examiner who performed all counts was not aware of the treatment received by the individual animals.

### 2.6 Experimental procedures

The study used 128 male Wistar rats in two experiments. For Experiment 1 we used 96 animals in 4 groups (n = 8); and for Experiment 2, we used 32 animals in 4 groups (n = 8). Each experimental group received a different pharmacological treatment.

#### 2.6.1 Experiment 1

**Figure 1:**
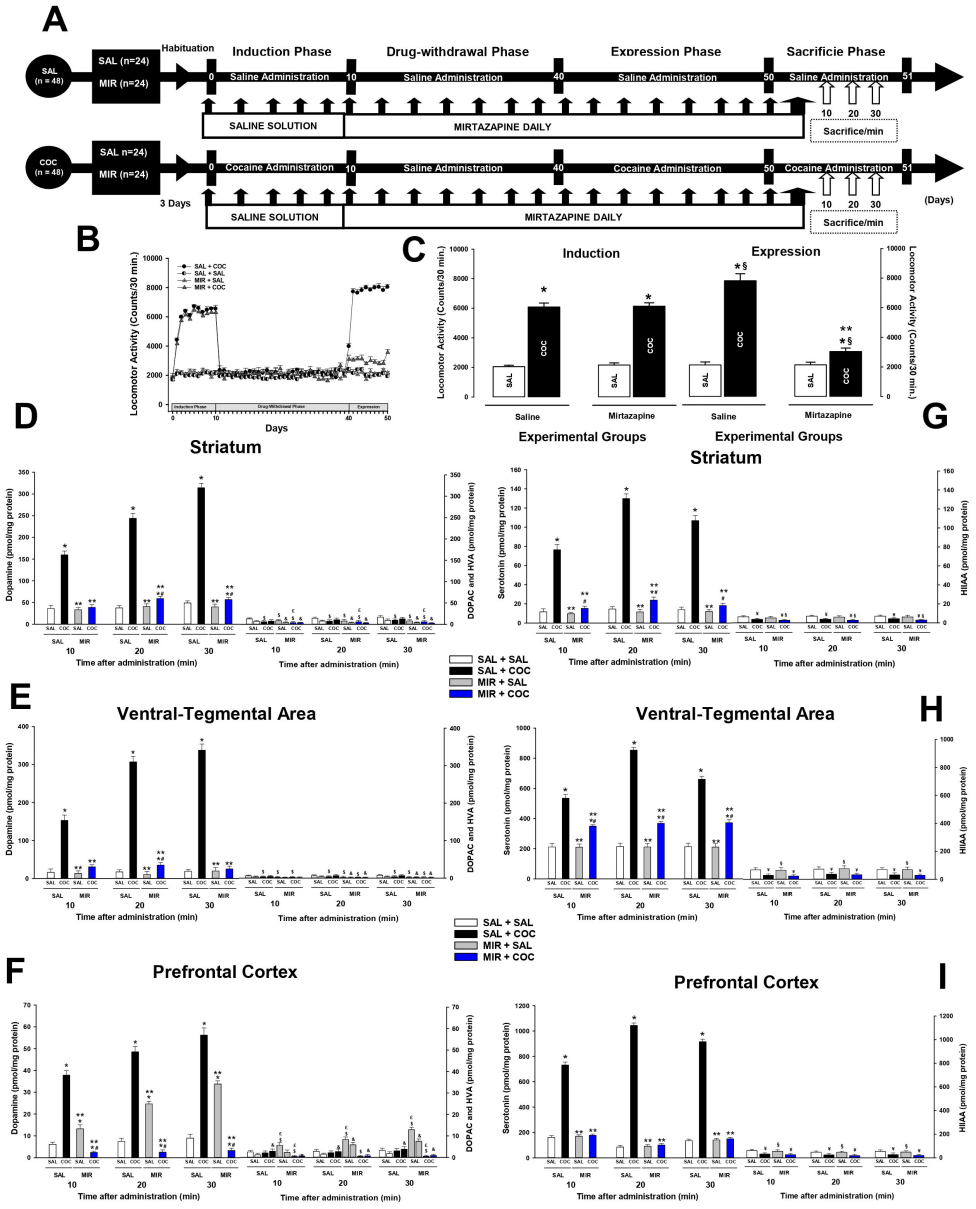
Mirtazapine reduces dopamine and serotonin levels. Experimental timeline. **(A)**. MIR (30 mg/kg i.p.) administered for 30 days during drug withdrawal attenuates cocaine-induced locomotor, and cocaine sensitization **(B, C)**. Mean locomotor activity (± S.E.M.) by group (n = 8 animals per group) *p < 0.01 significant effects of cocaine treatment on locomotor activity compared to the SAL + SAL groups. **p < 0.01 significant effects of different multitarget drugs on locomotor activity compared to the SAL + COC group, ^§^ p < 0.01 significant effects between the induction and expression phase, as determined by three-way ANOVA followed by Tukey’s tests. The dopamine and serotonin concentration (± S.E.M.) by group (n = 8 animals per group) in the striatum **(D, G)**, ventral-tegmental area **(E, H)**, and prefrontal cortex **(F, I)**. *p < 0.01 significant effects of cocaine treatment on dopamine or serotonin levels compared to the SAL + SAL groups. **p < 0.01 significant effects of mirtazapine on dopamine or serotonin levels compared to the SAL + COC group. ^#^p < 0.01 significant effects between the MIR + COC and MIR + SAL groups. ^$^p < 0.01 significant effects of cocaine treatment on DOPAC levels compared to the SAL + SAL groups. ^£^p < 0.01 significant effects of mirtazapine on DOPAC levels compared to the SAL + COC group. ^&^p < 0.01 significant effects of cocaine treatment on HVA levels compared to the SAL + SAL groups. ^¥^p < 0.01 significant effects of cocaine treatment on HIIAA levels compared to the SAL + SAL groups. ^§^p < 0.01 significant effects of mirtazapine on HIIAA levels compared to the SAL + COC group, as determined by four-way ANOVA followed by Tukey’s tests.

To determine the effect of mirtazapine on the cocaine-induced increase in dopamine levels, this experiment was divided into four experimental phases. Phase I, or the cocaine-induction phase, lasted 10 days. The drug-withdrawal phase lasted 30 days. Phase III, or the cocaine-expression phase, lasted 10 days. Phase IV, or the sacrifice phase, lasted 1 day (**Figure 1A**).

The SAL + SAL and SAL + MIR groups received saline, 30 minutes before saline daily administration, during the induction phase. During the drug-withdrawal, expression, and sacrifice phases received mirtazapine (30 mg/Kg), 30 minutes before saline administration.

The SAL + COC group received saline, 30 minutes before cocaine daily administration, during the induction, expression, and sacrifice phases. During the drug-withdrawal phase, cocaine was withdrawn, and the group received saline 30 minutes before saline administration.

The MIR + COC groups received cocaine daily during the induction phase. During the drug-withdrawal, expression, and sacrifice phases, the rats received mirtazapine, 30 minutes before the administration of saline or cocaine (10 mg/kg, i.p.), respectively. After each administration, the animals were immediately placed into the activity chambers, and the locomotor activity of each animal was recorded for 30 minutes.

In the sacrifice phase, after each administration, the animals were sacrificed at 10, 20, or 30 minutes after treatment (**Figure 1A**).

#### 2.6.2 Experiment 2

**Figure 2:**
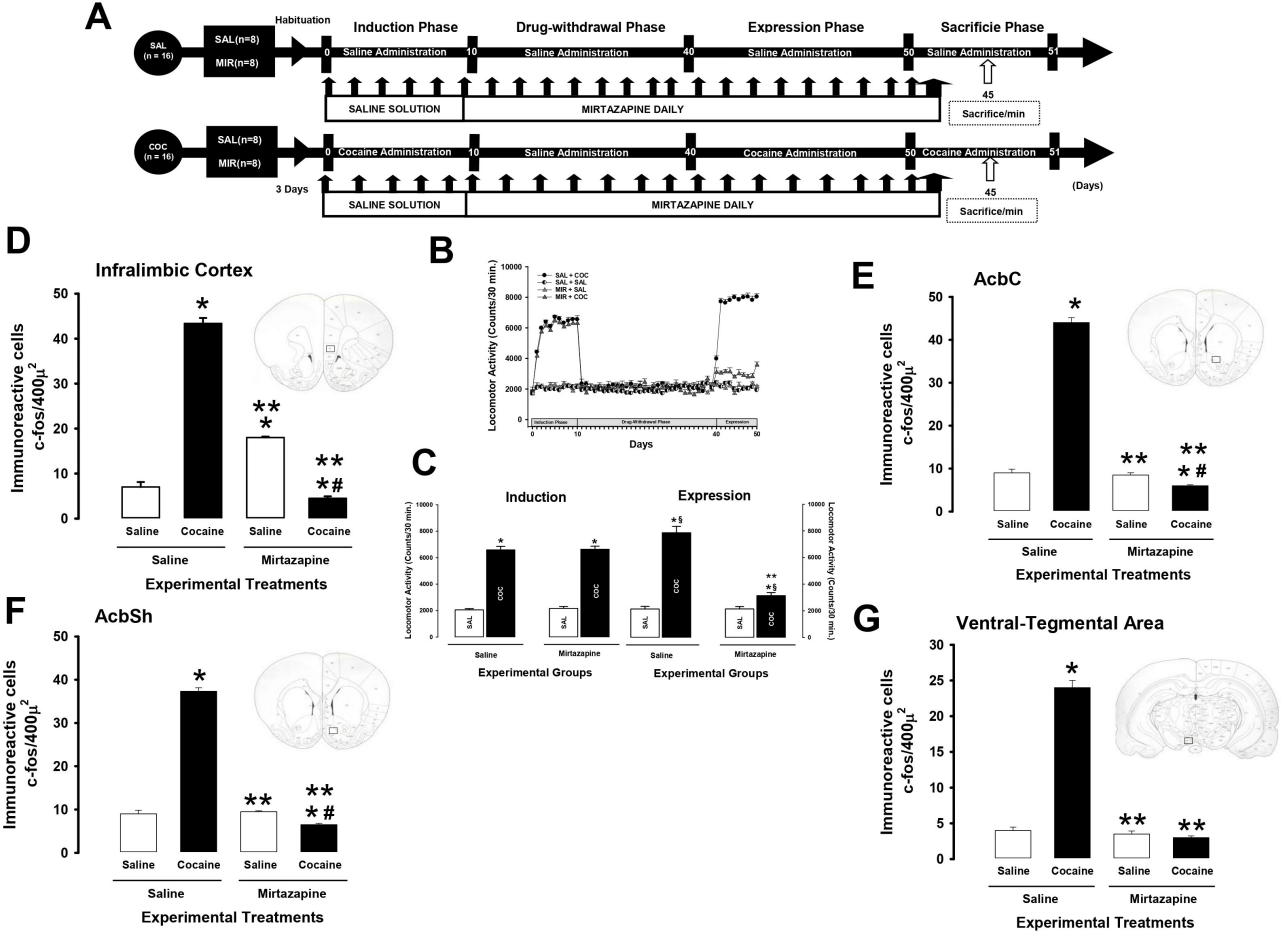
Mirtazapine reduces the number of cells immunoreactive to c-fos. Experimental timeline. **(A)**. MIR (30 mg/kg i.p.) administered for 30 days during drug withdrawal attenuates cocaine-induced locomotor, and cocaine sensitization **(B, C)**. Mean locomotor activity (± S.E.M.) by group (n = 8 animals per group) *p < 0.01 significant effects of cocaine treatment on locomotor activity compared to the SAL + SAL groups. **p < 0.01 significant effects of different multitarget drugs on locomotor activity compared to the SAL + COC group, ^§^ p < 0.01 significant effects between the induction and expression phase, as determined by three-way ANOVA followed by Tukey’s tests. The number of cells immunoreactive to c-fos (± S.E.M.) by group (n = 8 animals per group) in the infralimbic cortex **(D)**, AcbC **(E)**, AcbSh **(F)**, and ventral-tegmental area **(G)**. *p < 0.01 significant effects of cocaine treatment on the number of cells immunoreactive to c-fos compared to the SAL + SAL groups. **p < 0.01 significant effects of mirtazapine on the number of cells immunoreactive to c-fos compared to the SAL + COC group. ^#^p < 0.01 significant effects between the MIR + COC and MIR + SAL groups, as determined by two-way ANOVA followed by Tukey’s tests.

This experiment was performed to determine the effect of mirtazapine on cocaine-induced *Fos* protein expression. For this, Experiment 2 was divided into four phases. Phase I, or the cocaine-induction phase, lasted 10 days. Phase II, or the drug-withdrawal phase, lasted 30 days. The cocaine-expression phase lasted 10 days. Phase IV, or the sacrifice phase, lasted 1 day (**Figure 2A**).

The SAL+ SAL and SAL + MIR groups received saline solution (9% NaCl, i.p.), 30 minutes before saline daily administration, during the induction phase. During the drug-withdrawal, expression, and sacrifice phases received mirtazapine (30 mg/Kg), 30 minutes before saline administration.

The SAL + COC group received cocaine (10 mg/kg, i.p.) daily during induction and expression. During the cocaine-withdrawal phase, cocaine was withdrawn, and the groups received daily saline only.

The MIR + COC group received cocaine daily during the induction phase. During drug withdrawal and expression, the rats received mirtazapine 30 minutes before administration of either saline or cocaine (10 mg/kg, i.p.).

During the sacrifice phase, the SAL + SAL, SAL + MIR, SAL + COC and MIR + COC groups received saline (9% NaCl, i.p.) or mirtazapine (30 mg/kg, i.p.), 30 minutes before saline or cocaine (10 mg/kg, i.p.), respectively. After each administration, the animals were sacrificed 45 minutes after treatment. (**Figure 2A**). The intracellular cascade of events to produce peak levels of the protein c-fos is estimated to take ∼45 min (Wang et al., 1996).

### 2.7 Statistical analysis

Data are expressed as the means ± S.E.M. Locomotor activity was measured by counting beam breaks during the testing session. For the graphic representation, in

In experiments 1 and 2, to determine the effect of each of the mirtazapine on cocaine locomotor sensitization, the mean cocaine-induced locomotor activity of the last 10 days of the induction phase was compared versus the mean locomotor activity of the first 10 days of the expression phase (comparison between phases). The results for locomotor activity in each group during the expression phase were analyzed with a three-way analysis of variance (ANOVA) with treatments (saline, or mirtazapine), groups (saline or cocaine), and phase (induction and expression) as the between-subjects factors. If the interaction yielded a significant F value, a *post-hoc* analysis of differences was performed between groups followed by an additional Tukey’s test.

For Experiment 1, we used a four-way ANOVA with groups, treatments, time (10, 20, 30 minutes), and metabolites as the between-subject factors. For Experiment 2, the study used a two-way analysis of variance (ANOVA) with treatments (saline, or mirtazapine) and groups (saline or cocaine) as the between-subject factors, followed by a *post-hoc* analysis. When there was a significant F value in the interaction, a Tukey test of differences between groups was performed. Statistical analysis was performed with SPSS version 21 (IBM, 2021). The statistical significance level was set at p < 0.05.

## 3 Results

### 3.1 Locomotor activity

As shown in **Figures 1B, C**, **2B, C**, cocaine significantly increased locomotor activity during expression (Experiment 1; three-way ANOVA; in the group X treatment X phase interaction, F (1, 64) = 269.040 p < 0.0001 experiments 2; three-way ANOVA; in the group X treatment X phase interaction, F (1, 64) = 271.538 p < 0.0001), compared to the SAL + SAL (p < 0.0001) and the MIR + SAL (p < 0.0001) groups. In contrast, in rats that had previously received a dose of mirtazapine during cocaine withdrawal, cocaine administration did not significantly increase locomotor activity, as occurred in animals in the SAL + COC group. The Tukey’s test found differences in cocaine-induced locomotor activity when comparing the MIR + COC group to the SAL + SAL (p < 0.002), the MIR + SAL (p < 0.002), and the SAL + COC (p < 0.0001) groups (**Figures 1B**, **2B**).

When the differences between the induction phase and the expression phase (locomotor sensitization) were compared, Tukey’s test found significant differences in the cocaine-induced locomotor activity shown during the induction phase compared to that shown in the expression phase in the SAL + COC (p < 0.001) group. Additionally, the *post-hoc* test found a decrease in cocaine-induced locomotor activity during the induction phase compared to that shown during the expression phase in the MIR + COC (p < 0.001) groups. This suggests that the treatment decreased the expression of cocaine locomotor sensitization.

### 3.2 Experiment 1

#### 3.2.1 Dopamine

**Figures 1D–F** shows the dopamine levels in the striatum (four-way ANOVA; in the groups, treatments, time X metabolite interaction, F (4, 288) = 1307.358 p < 0.0001), PFC (four-way ANOVA; in the groups, treatments, time X metabolite interaction, F (4, 288) = 6623.183 p < 0.0001) and VTA (four-way ANOVA; in the groups, treatments, time X metabolite interaction, F (4, 288) = 134.799 p < 0.0001) at 10, 20 or 30 minutes after the administration of the treatments.

The statistical analysis found differences in the levels of dopamine shown by the animals of the SAL + COC group compared to those shown by the SAL + SAL (p < 0.0001) and MIR + SAL (p < 0.0001) groups at 10, 20, or 30 minutes after administration in each of the brain structures analyzed (**Figures 1D–F**).

Tukey’s test found significant differences in the levels of dopamine in the striatum, PFC, and VTA between the MIR + COC group and the SAL + COC (p < 0.0001) group, at 10, 20, or 30 minutes after administration. However, there were no differences between the MIR + COC and MIR + SAL groups (p = 0.81) in the VTA.

Furthermore, the *post-hoc* test found significant differences in the levels of DOPAC in the striatum, VTA, and PFC showed by the SAL + SAL group concerning the levels shown by the MIR + SAL (p < 0.002) and MIR + COC groups (p < 0.002) at 10, 20, or 30 minutes after administration. In addition, Tukey’s test revealed differences between the SAL + COC group concerning the MIR + SAL (p < 0.002) and MIR + COC (p < 0.002) groups in the striatum and PFC, but the *post-hoc* analysis did not find differences between the SAL + COC group concerning the MIR + SAL (p = 0.64) and MIR + COC (p = 0.71) groups in the VTA at 10, 20, or 30 minutes after administration.

Regarding HVA levels, Tukey’s test found differences between the SAL + COC group concerning the MIR + SAL (p < 0.002) and MIR + COC (p < 0.002) groups in the striatum and PFC, but it did not find differences between the SAL + COC group concerning the MIR + SAL (p = 0.59) and MIR + COC (p = 0.78) groups in the VTA at 10, 20, or 30 minutes after administration.

#### 3.2.2 Serotonin

**Figures 1G–I** shows the serotonin levels in the striatum (four-way ANOVA; in the groups, treatments, time X metabolite interaction, F (2, 191) = 756.174 p < 0.0001), PFC (four-way ANOVA; in the groups, treatments, time X metabolite interaction, F (2, 191) = 26224.651 p < 0.0001) and VTA (four-way ANOVA; in the groups, treatments, time X metabolite interaction, F (2, 191) = 29806.773 p < 0.0001) at 10, 20 or 30 minutes after the administration of the treatments.

The statistical analysis found differences in the levels of serotonin shown by the animals of the SAL + COC group compared to those shown by the SAL + SAL (p < 0.0001) and MIR + SAL (p < 0.0001) groups at 10, 20, or 30 minutes after administration in each of the brain structures analyzed (**Figures 1G–I**).

Tukey’s test found significant differences in the levels of serotonin in the striatum, PFC, and VTA between the MIR + COC group and the SAL + COC (p < 0.0001) group, at 10, 20, or 30 minutes after administration. Furthermore, the *post-hoc* test found differences between the MIR + SAL and MIR + COC (p < 0.002) groups in the levels of serotonin in the striatum and VTA. However, he found no differences (p = 0.94) between these groups in the PFC at 10, 20, or 30 minutes after administration.

However, there were no differences between the SAL + SAL and MIR + SAL groups (p = 0.97) in the striatum, PFC, and VTA, at 10, 20, or 30 minutes after administration.

Additionally, Tukey’s test revealed differences in the levels of HIIAA in the striatum, PFC, and VTA shown by the SAL + SAL group compared to those shown by the SAL + COC (p < 0.0002) and MIR + COC (p < 0.0002) groups. But no differences were found concerning the MIR + SAL (p = 0.92) group, at 10, 20, or 30 minutes after administration. Furthermore, the statistical test revealed differences in the levels of HIIAA in the PFC, and VTA shown by the SAL + COC group compared to the MIR + SAL (p < 0.002) group but did not find differences concerning the MIR + COC (p = 0.96) group, at 10, 20, or 30 minutes after administration.

In the striatum, Tukey’s test revealed differences in HIIAA levels, at 10, 20, or 30 minutes after administration, between the SAL + COC and MIR + COC (p < 0.002) groups. However, the *post-hoc* test did not find differences concerning the MIR + SAL (p = 0.87) group.

### 3.3 Experiment 2

Two-way ANOVA found significant differences in the number of c-fos-IR cells in the IL (two-way ANOVA; in the groups by treatment interaction, F (1, 31) = 3245.773 p < 0.0001), AcbSh (two-way ANOVA; in the groups by treatment interaction, F (1. 31) = 1582.824 p < 0.0001), AcbC (two-way ANOVA; in the groups by treatment interaction, F (1. 31) = 1968.75 p < 0.0001), and VTA (two-way ANOVA; in the groups by treatment interaction, F (1. 31) = 653.722 p < 0.0001).

**Figure 3:**
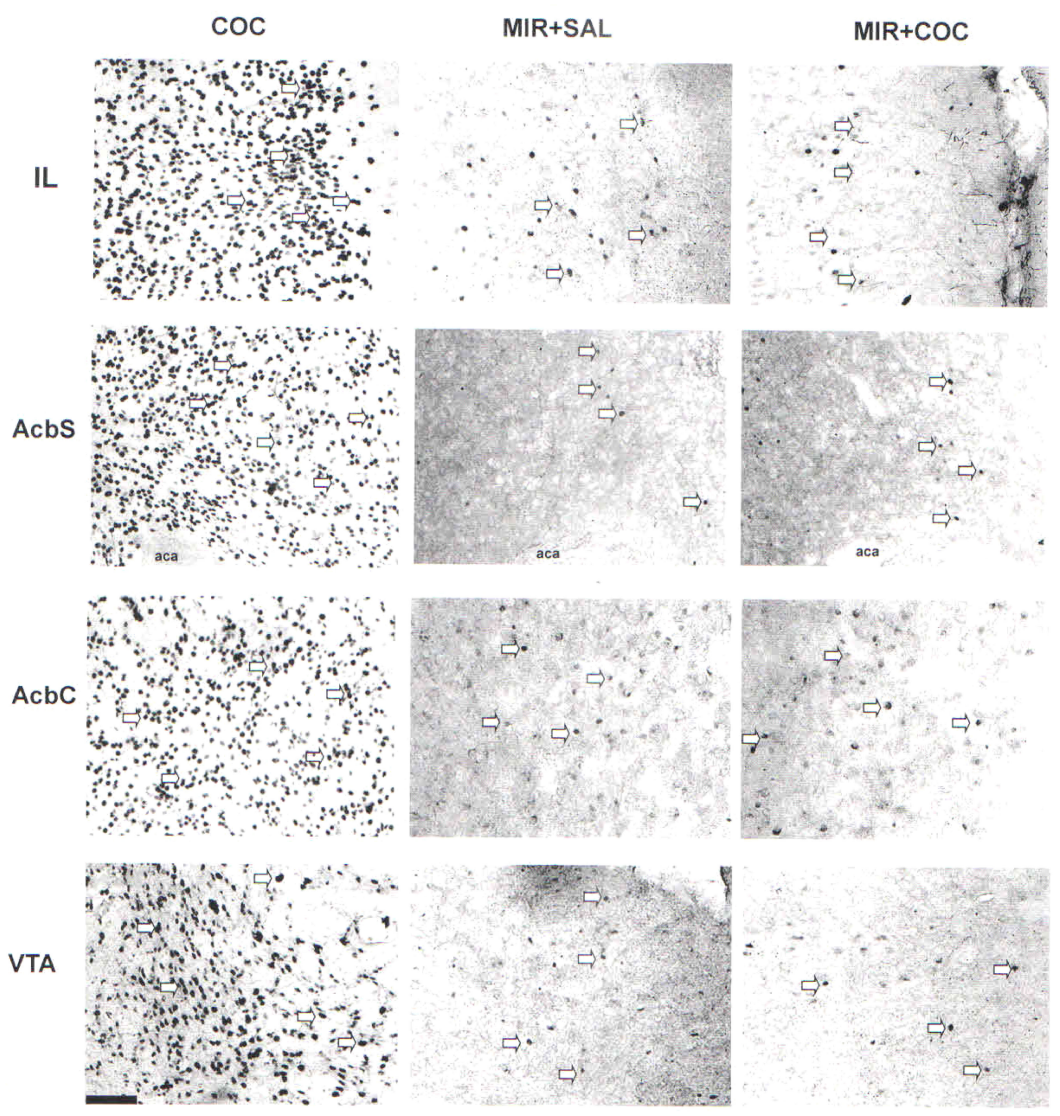
Representative photomicrographs of Fos protein expression in the IL, AcbS, AcbC, and VTA of rats from all experimental groups at 20× magnification, where Fos protein expression was visible as dark ovals (highlighted by arrows). Scale bar = 100 μm.

Tukey’s test found differences in the number of c-fos-IR cells as shown by the animals of the SAL + COC group compared to those shown by the SAL + SAL (p < 0.0001), MIR + SAL (p < 0.0001), and MIR + SAL (p < 0.0001) groups in each of the brain nuclei analyzed (**Figures 2D–G**, **3**).

The *post-hoc* test found significant differences in the number of c-fos-IR cells in the IL, AcbSh, AcbC, and VTA between the MIR + COC group and the MIR + SAL (p < 0.0001) group (**Figures 2D–G**, **3**).

## 4 Discussion

Pioneering studies demonstrated a cocaine-induced increase in dopamine and serotonin release in the ventral striatum, VTA, and PFC (4, 5, 51–57). The results of this study are in line with this evidence, where cocaine administration generated an increase in dopamine and serotonin levels in the ventral striatum, VTA, and PFC.

On the other hand, several studies have revealed that cocaine-induced behavioral sensitization is related to a rise in dopamine levels (58, 59), which reaches its maximum level at 20–40 minutes of injection and returns to baseline levels 80 minutes after injection (60, 61). These results are consistent with what was observed in this study, where the locomotor activity induced by cocaine is associated with an increase in dopamine levels in the Acb, VTA, and PFC.

However, the magnitude of the cocaine-induced increase in dopamine levels differs from that reported by other studies (4, 62–65). Various studies have shown that cocaine-induced stimulant actions involve cocaine-induced joint alteration of various neurotransmission systems (66, 67). Cocaine acts on the neuronal membrane monoamine transporters NET, DAT, and SERT to inhibit the reuptake of neurotransmitters. However, the psychostimulant effects of cocaine may have additional effects involving pre- and postsynaptic/junctional receptors for norepinephrine, serotonin, and other receptors. As a result, cocaine’s psychostimulant effects can be greatly increased. Pioneering studies have reported that cocaine increases the levels of 5-HT and NE (54–57) in the mesolimbic-cortical system. These studies have shown that 5-HT and NE modulate cocaine-induced dopamine levels, through their action on 5-HT_2_, 5-HT_3_, and α_1_NE receptors located on dopaminergic neurons (68, 69). Thus, activation of these receptors enhances the increase in cocaine-induced dopamine levels in the ventral striatum and PFC (68, 70, 71). On the other hand, other studies have shown that cocaine induces an increase in the levels of glutamate in the prefrontal cortex and in the ventral tegmental area (71, 72), which, through its action on mGlu2/3/5 receptors, modulates dopamine levels in the ventral striatum (73–76). Thus, the joint action of these three neurotransmission systems and probably others (cannabinoids) on the dopaminergic neurons of the ventral striatum and the PFC, could explain the increase in dopamine levels reported in this study.

Other studies have shown that mirtazapine alters dopamine levels in freely moving rats via the blockade of α_2_-adrenergic receptors and agonism of postsynaptic 5-HT**_1A_** receptors (77–80) in the PFC. These studies additionally reported that mirtazapine could not increase intracellular dopamine levels in the ventral striatum or VTA. These results are consistent with what was found in this study, where mirtazapine only increased dopamine levels in the PFC.

Furthermore, in this study we found that mirtazapine slightly affected DOPAC and HVA levels in the striatum and VTA; but in the PFC, mirtazapine increased the levels of DOPAC and HVA. These results agree with previous works that show that mirtazapine did not alter the levels of DOPAC and HVA in the striatum but increased them in the PFC (78, 81).

Studies in rodents reported that the administration of mirtazapine did not generate an increase in 5-HT levels in PFC and striatum (77, 79). Our results are in line with these studies. Where the administration of mirtazapine did not increase the release of 5-HT in the striatum, VTA, and PFC.

We found that mirtazapine dosing significantly decreased cocaine-induced ex vivo relative DA and 5-HT content in the ventral striatum, VTA, and PFC. We have not found similar results in the literature. However, individual dosing of serotonin 5-HT_2A_ or 5-HT_3_ receptor antagonists have been reported (67, 82–85) or α_2_ NE and serotonin 5-HT_1A_ receptor agonists (54, 86, 87) decreased cocaine-induced dopamine levels. These results suggest that the mirtazapine-induced decrease in cocaine-induced DA and 5-HT levels is probably due to the action of mirtazapine on pre- and post-synaptic 5-HT_1A_, 5-HT_2A_, and 5-HT3 receptors located on dopaminergic and serotonergic neurons.

Pioneering studies showed that cocaine induces an increase in immunoreactivity to c-fos in the ventral striatum (88, 89). Similar results were found in this study, where 10 mg/kg cocaine significantly increased the number of c-fos immunoreactive cells in the ventral striatum, VTA, and PFC.

In contrast, mirtazapine dosing significantly decreased cocaine-induced c-fos expression. To our knowledge, similar results have not been reported. However, the blockade of serotonin 5-HT_2A_ or 5-HT_3_ receptors and activation of serotonin 5-HT**_2C_** receptors reduced cocaine-induced *Fos* protein expression in the striatum (82, 90–92).

Some studies have reported that the cocaine-induced increase in dopamine levels is related to the increase in immunoreactivity to c-fos in the striatum (11, 12, 91). These studies showed that cocaine-induced *Fos* protein expression depends on differential activation of dopamine D_1_ and D_2_ receptors (11, 12, 93).

Thus, like the increase in cocaine-induced locomotor activity and the increase in cocaine-induced *Fos* protein expression, it is related to the increase in cocaine-induced dopamine levels and depends on the differential activation of dopamine D_1_ and D_2_ receptors in the striatum. Other studies have shown that mirtazapine produced altered expression in the ratio of D1/D2-like dopamine receptors in the NAcc, which could lead to a decrease in dopamine levels and subsequent attenuation of cocaine-induced locomotor sensitization and c-fos protein expression (94, 95).

In summary, 1) cocaine increases the levels of DA (4, 5, 51–53), 5-HT (54–57), and the expression of the c-fos protein in the mesolimbic-cortical system. 2) the activation of 5-HT_2_, 5-HT_3_, and α_1_ NE receptors located on dopaminergic neurons enhances the increase in cocaine-induced dopamine levels (68–71). 3) Serotonin 5-HT_2A_ or 5-HT_3_ receptor antagonists (67, 82–85) or α_2_ NE and serotonin 5-HT_1A_ receptor agonists decreased cocaine-induced dopamine levels (54, 86, 87) and *Fos* protein expression in the striatum (82, 90–92). 4) Mirtazapine, carries out its therapeutic effects through antagonism of the α_2_ NE receptor, and block of the 5HT_2A/C_ and 5HT_3_ and histamine 1 (H_1_R) receptors (29). Additionally, mirtazapine can also act as an inverse agonist of the 5-HT_2C_ receptor and indirectly as an agonist of the 5-HT_1A_ receptor (80, 96). Thus, it is likely that mirtazapine, by simultaneously antagonizing 5-HT_2A_ and 5-HT_3_ receptors and activating 5-HT_1A_ receptors, decreased cocaine-induced DA and 5-HT levels, the activation of dopamine D_1_ and D_2_ receptors, and the subsequent expression of the Fos protein in the mesolimbic-cortical system.

## 5 Conclusions

The cocaine-induced locomotor activity depends mainly on the increase in extracellular levels of dopamine and on the subsequent activation of different neuronal circuits (4, 88). Additionally, the effects on behavior (expression of locomotor sensitization) and synaptic reorganization produced by chronic cocaine exposure depend on the dopamine-dependent Fos protein expression (22, 23). On the other hand, previously we demonstrated that the daily dosage of mirtazapine during withdrawal decreased the induction and expression of locomotor sensitization to methamphetamine, cocaine, and nicotine (27, 32, 33, 39) in male rats. Additionally, in this study, we showed for the first time that mirtazapine significantly attenuated the cocaine-induced increase in dopamine release and Fos protein expression. Thus, these data suggest that mirtazapine probably, through its mechanism of action, decreased the cocaine-induced increase in dopamine levels, which resulted in a decrease in the activation of dopamine D_1_ receptors and the subsequent cocaine-induced *Fos* protein expression. This could prevent the consolidation of changes in behavior (decreased mirtazapine-dependent expression of cocaine locomotor sensitization) and in the cocaine-induced reorganization of neuronal circuits. It would explain the mirtazapine-induced effects on cocaine behavioral sensitization. Thus, these data together could support its possible use for the treatment of patients with CUD.

## Data Availability

The datasets presented in this study can be found in online repositories. The names of the repository/repositories and accession number(s) can be found in the article/**Supplementary Material**.
