# Supplementary material for: Mirtazapine decreased cocaine-induced c-fos expression and dopamine release in rats
Source: Front Psychiatry. 2024 Aug 12;15:1428730. doi: 10.3389/fpsyt.2024.1428730 (PMC11346032; doi:10.3389/fpsyt.2024.1428730)
Supplement: Supplementary file 1 [file DataSheet_1.pdf]

EXPERIMENT 1

Dopamine-Striatum

Estadísticos descriptivos

Variable dependiente: HPLC

Grupos	Tiempos	Dopamina	Media	Desviación típica	N
1.00	1.00	1.00	36.1625	2.33403	8
		2.00	12.9375	1.08356	8
		3.00	6.8750	1.24642	8
		Total	18.6583	12.98995	24
	2.00	1.00	37.4500	2.13809	8
		2.00	14.2500	1.03510	8
		3.00	7.5000	.92582	8
		Total	19.7333	13.17823	24
	3.00	1.00	49.0000	1.19523	8
		2.00	17.0000	1.30931	8
		3.00	9.5000	.92582	8
		Total	25.1667	17.53175	24
	Total	1.00	40.8708	6.18471	24
		2.00	14.7292	2.04822	24
		3.00	7.9583	1.51741	24
		Total	21.1861	14.78909	72
2.00	1.00	1.00	159.8750	1.95941	8
		2.00	5.8750	1.24642	8
		3.00	7.3750	.74402	8
		Total	57.7083	73.81144	24
	2.00	1.00	243.8000	2.77746	8
		2.00	7.3750	.74402	8
		3.00	10.7500	.70711	8
		Total	87.3083	113.05686	24
	3.00	1.00	314.0000	3.38062	8
		2.00	9.3750	1.18773	8
		3.00	12.8750	.99103	8
		Total	112.0833	145.86918	24
	Total	1.00	239.2250	64.41378	24
		2.00	7.5417	1.79320	24
		3.00	10.3333	2.44357	24
		Total	85.7000	115.32271	72
3.00	1.00	1.00	33.2500	1.88982	8
		2.00	8.6250	.91613	8
		3.00	4.3750	.74402	8
		Total	15.4167	13.06062	24
	2.00	1.00	40.6500	1.06904	8
		2.00	7.8750	.83452	8
		3.00	3.6250	.51755	8
		Total	17.3833	16.91803	24
	3.00	1.00	39.7250	1.67396	8
		2.00	7.8750	.35355	8
		3.00	3.8750	.35355	8
		Total	17.1583	16.41370	24
	Total	1.00	37.8750	3.68738	24
		2.00	8.1250	.79741	24
		3.00	3.9583	.62409	24
		Total	16.6528	15.36340	72
4.00	1.00	1.00	38.7250	1.00960	8
		2.00	3.8750	.64087	8
		3.00	3.7500	.70711	8
		Total	15.4500	16.82943	24
	2.00	1.00	59.0000	3.70328	8
		2.00	5.8750	1.24642	8
		3.00	3.0000	.53452	8
		Total	22.6250	26.39139	24
	3.00	1.00	56.7000	1.19523	8
		2.00	5.6250	.74402	8
		3.00	2.8750	.83452	8
		Total	21.7333	25.29909	24
	Total	1.00	51.4750	9.52125	24
		2.00	5.1250	1.26190	24
		3.00	3.2083	.77903	24
		Total	19.9361	23.13127	72
Total	1.00	1.00	67.0031	54.54184	32
		2.00	7.8281	3.57998	32
		3.00	5.5938	1.79353	32
		Total	26.8083	42.34519	96
	2.00	1.00	95.2250	87.58688	32
		2.00	8.8438	3.38983	32
		3.00	6.2188	3.25016	32
		Total	36.7625	65.10451	96
	3.00	1.00	114.8563	116.99141	32
		2.00	9.9688	4.43944	32
		3.00	7.2813	4.23682	32
		Total	44.0354	83.74950	96
	Total	1.00	92.3615	91.26961	96
		2.00	8.8802	3.89044	96
		3.00	6.3646	3.29312	96
		Total	35.8688	66.09308	288

Pruebas de los efectos inter-sujetos

Variable dependiente: HPLC

Origen	Suma de cuadrados tipo III	gl	Media cuadrática	F	Sig.	Eta al cuadrado parcial	Parámetro de no centralidad Parámetro	Potencia observada[b]
Modelo corregido	1253158,26[a]	35	35804.522	16632.468	.000	1.000	582136.366	1.000
Intersección	370531.361	1	370531.361	172124.932	.000	.999	172124.932	1.000
Grupos	239172.187	3	79724.062	37034.649	.000	.998	111103.947	1.000
Tiempos	14360.101	2	7180.050	3335.388	.000	.964	6670.775	1.000
Dopamina	459869.119	2	229934.560	106812.742	.000	.999	213625.483	1.000
Grupos * Tiempos	22586.653	6	3764.442	1748.717	.000	.977	10492.300	1.000
Grupos * Dopamina	455516.881	6	75919.480	35267.286	.000	.999	211603.715	1.000
Tiempos * Dopamina	22792.202	4	5698.050	2646.946	.000	.977	10587.784	1.000
Grupos * Tiempos * Dopamina	38861.113	12	3238.426	1504.364	.000	.986	18052.363	1.000
Error	542.478	252	2.153					
Total	1624232.095	288						
Total corregida	1253700.734	287						

a. R cuadrado = 1.000 (R cuadrado corregida = 1.000)

b. Calculado con alfa = .05

EXPERIMENT 1

Dopamine-PFC

Estadísticos descriptivos

Variable dependiente: HPLC

Grupos	Tiempos	Dopamina	Media	Desviación típica	N
1.00	1.00	1.00	16.1500	1.29615	8
		2.00	5.8750	.64087	8
		3.00	3.3750	.74402	8
		Total	8.4667	5.71768	24
	2.00	1.00	17.4875	1.21354	8
		2.00	6.1250	.35355	8
		3.00	3.6250	.51755	8
		Total	9.0792	6.20820	24
	3.00	1.00	19.0000	1.51186	8
		2.00	7.5000	.92582	8
		3.00	3.5000	.75593	8
		Total	10.0000	6.79514	24
	Total	1.00	17.5458	1.75201	24
		2.00	6.5000	.97802	24
		3.00	3.5000	.65938	24
		Total	9.1819	6.19969	72
2.00	1.00	1.00	152.8750	1.35620	8
		2.00	3.2500	.46291	8
		3.00	4.5000	.75593	8
		Total	53.5417	71.75743	24
	2.00	1.00	306.8750	1.55265	8
		2.00	3.6250	.74402	8
		3.00	6.3750	.74402	8
		Total	105.6250	145.37412	24
	3.00	1.00	337.5000	1.06904	8
		2.00	3.7500	.70711	8
		3.00	6.5000	.92582	8
		Total	115.9167	160.05948	24
	Total	1.00	265.7500	82.53550	24
		2.00	3.5417	.65801	24
		3.00	5.7917	1.21509	24
		Total	91.6944	132.54897	72
3.00	1.00	1.00	13.2550	1.72689	8
		2.00	2.0000	.53452	8
		3.00	1.3750	.51755	8
		Total	5.5433	5.67201	24
	2.00	1.00	10.7500	.70711	8
		2.00	1.8750	.64087	8
		3.00	1.1250	.35355	8
		Total	4.5833	4.50040	24
	3.00	1.00	19.8750	1.35620	8
		2.00	1.8750	.35355	8
		3.00	2.0000	.00000	8
		Total	7.9167	8.67238	24
	Total	1.00	14.6267	4.13306	24
		2.00	1.9167	.50361	24
		3.00	1.5000	.51075	24
		Total	6.0144	6.58310	72
4.00	1.00	1.00	30.7500	2.31455	8
		2.00	2.1250	.35355	8
		3.00	1.1250	.35355	8
		Total	11.3333	14.09183	24
	2.00	1.00	35.2500	2.25198	8
		2.00	1.8750	.64087	8
		3.00	1.1250	.35355	8
		Total	12.7500	16.30751	24
	3.00	1.00	25.0000	1.30931	8
		2.00	1.5000	.53452	8
		3.00	1.0000	.00000	8
		Total	9.1667	11.46513	24
	Total	1.00	30.3333	4.69659	24
		2.00	1.8333	.56466	24
		3.00	1.0833	.28233	24
		Total	11.0833	13.97357	72
Total	1.00	1.00	53.2575	58.84430	32
		2.00	3.3125	1.65466	32
		3.00	2.5938	1.54208	32
		Total	19.7213	41.23025	96
	2.00	1.00	92.5906	126.03367	32
		2.00	3.3750	1.86219	32
		3.00	3.0625	2.25671	32
		Total	33.0094	83.54529	96
	3.00	1.00	100.3438	139.13827	32
		2.00	3.6563	2.49657	32
		3.00	3.2500	2.18499	32
		Total	35.7500	91.80987	96
	Total	1.00	82.0640	114.28023	96
		2.00	3.4479	2.02026	96
		3.00	2.9688	2.01809	96
		Total	29.4935	75.58018	288

Pruebas de los efectos inter-sujetos

Variable dependiente: HPLC

Origen	Suma de cuadrados tipo III	gl	Media cuadrática	F	Sig.	Eta al cuadrado parcial	Parámetro de no centralidad Parámetro	Potencia observada[b]
Modelo corregido	1639191,99[a]	35	46834.057	46044.943	.000	1.000	1611573.022	1.000
Intersección	250522.272	1	250522.272	246301.188	.000	.999	246301.188	1.000
Grupos	372363.629	3	124121.210	122029.874	.000	.999	366089.623	1.000
Tiempos	14112.196	2	7056.098	6937.209	.000	.982	13874.417	1.000
Dopamina	397976.435	2	198988.217	195635.437	.000	.999	391270.875	1.000
Grupos * Tiempos	39887.916	6	6647.986	6535.973	.000	.994	39215.839	1.000
Grupos * Dopamina	711362.010	6	118560.335	116562.696	.000	1.000	699376.173	1.000
Tiempos * Dopamina	26690.016	4	6672.504	6560.078	.000	.990	26240.312	1.000
Grupos * Tiempos * Dopamina	76799.794	12	6399.983	6292.149	.000	.997	75505.783	1.000
Error	256.319	252	1.017					
Total	1889970.585	288						
Total corregida	1639448.313	287						

a. R cuadrado = 1.000 (R cuadrado corregida = 1.000)

b. Calculado con alfa = .05

EXPERIMENT 1

Dopamine-VTA

Estadísticos descriptivos

Variable dependiente: HPLC

Grupos	Tiempos	Dopamina	Media	Desviación típica	N
1.00	1.00	1.00	6.1250	1.45774	8
		2.00	2.5000	.53452	8
		3.00	1.2500	.46291	8
		Total	3.2917	2.29326	24
	2.00	1.00	7.5000	1.19523	8
		2.00	2.8750	.83452	8
		3.00	1.5000	.53452	8
		Total	3.9583	2.75806	24
	3.00	1.00	8.8750	.83452	8
		2.00	3.2500	.88641	8
		3.00	1.8750	.83452	8
		Total	4.6667	3.19873	24
	Total	1.00	7.5000	1.61515	24
		2.00	2.8750	.79741	24
		3.00	1.5417	.65801	24
		Total	3.9722	2.79321	72
2.00	1.00	1.00	37.8750	2.10017	8
		2.00	2.0000	.53452	8
		3.00	2.8750	1.12599	8
		Total	14.2500	17.12169	24
	2.00	1.00	49.0000	1.30931	8
		2.00	2.1250	.83452	8
		3.00	2.1250	.35355	8
		Total	17.7500	22.58944	24
	3.00	1.00	56.5000	2.44949	8
		2.00	3.0000	.75593	8
		3.00	3.8750	.83452	8
		Total	21.1250	25.59775	24
	Total	1.00	47.7917	8.04865	24
		2.00	2.3750	.82423	24
		3.00	2.9583	1.08264	24
		Total	17.7083	21.92075	72
3.00	1.00	1.00	13.2500	1.66905	8
		2.00	5.6250	1.06066	8
		3.00	2.3750	.74402	8
		Total	7.0833	4.79961	24
	2.00	1.00	25.1250	1.24642	8
		2.00	8.3750	.74402	8
		3.00	5.8750	.83452	8
		Total	13.1250	8.77899	24
	3.00	1.00	33.8750	.83452	8
		2.00	12.8750	.83452	8
		3.00	7.5000	.92582	8
		Total	18.0833	11.65413	24
	Total	1.00	24.0833	8.72237	24
		2.00	8.9583	3.16886	24
		3.00	5.2500	2.32659	24
		Total	12.7639	9.84599	72
4.00	1.00	1.00	2.3750	.91613	8
		2.00	.3750	.51755	8
		3.00	.6250	.74402	8
		Total	1.1250	1.15392	24
	2.00	1.00	2.5000	.53452	8
		2.00	.5000	.75593	8
		3.00	.7500	.70711	8
		Total	1.2500	1.11316	24
	3.00	1.00	3.3750	1.59799	8
		2.00	.5000	.53452	8
		3.00	.8750	.35355	8
		Total	1.5833	1.61290	24
	Total	1.00	2.7500	1.15156	24
		2.00	.4583	.58823	24
		3.00	.7500	.60792	24
		Total	1.3194	1.30898	72
Total	1.00	1.00	14.9063	14.12698	32
		2.00	2.6250	2.04387	32
		3.00	1.7813	1.18415	32
		Total	6.4375	10.16373	96
	2.00	1.00	21.0313	18.52546	32
		2.00	3.4688	3.10031	32
		3.00	2.5625	2.09358	32
		Total	9.0208	13.76875	96
	3.00	1.00	25.6563	21.58645	32
		2.00	4.9063	4.85506	32
		3.00	3.5313	2.67587	32
		Total	11.3646	16.29732	96
	Total	1.00	20.5313	18.67503	96
		2.00	3.6667	3.61770	96
		3.00	2.6250	2.17764	96
		Total	8.9410	13.74507	288

Pruebas de los efectos inter-sujetos

Variable dependiente: HPLC

Origen	Suma de cuadrados tipo III	gl	Media cuadrática	F	Sig.	Eta al cuadrado parcial	Parámetro de no centralidad Parámetro	Potencia observada[b]
Modelo corregido	53952.622[a]	35	1541.503	1442.075	.000	.995	50472.615	1.000
Intersección	23023.003	1	23023.003	21537.993	.000	.988	21537.993	1.000
Grupos	12546.538	3	4182.179	3912.424	.000	.979	11737.272	1.000
Tiempos	1166.174	2	583.087	545.477	.000	.812	1090.954	1.000
Dopamina	19396.257	2	9698.128	9072.588	.000	.986	18145.176	1.000
Grupos * Tiempos	883.160	6	147.193	137.699	.000	.766	826.195	1.000
Grupos * Dopamina	18509.826	6	3084.971	2885.987	.000	.986	17315.921	1.000
Tiempos * Dopamina	829.160	4	207.290	193.919	.000	.755	775.678	1.000
Grupos * Tiempos * Dopamina	621.507	12	51.792	48.452	.000	.698	581.419	1.000
Error	269.375	252	1.069					
Total	77245.000	288						
Total corregida	54221.997	287						

a. R cuadrado = .995 (R cuadrado corregida = .994)

b. Calculado con alfa = .05

Estadísticos descriptivos

Variable dependiente: HPLC

Grupos	Tiempos	Serotonin	Media	Desviación típica	N
1.00	1.00	1.00	11.6250	.74402	8
		2.00	6.3750	.74402	8
		Total	9.0000	2.80476	16
	2.00	1.00	14.5000	.75593	8
		2.00	6.8750	.83452	8
		Total	10.6875	4.01196	16
	3.00	1.00	13.8571	.69007	7
		2.00	6.8750	1.12599	8
		Total	10.1333	3.71996	15
	Total	1.00	13.3043	1.45960	23
		2.00	6.7083	.90790	24
		Total	9.9362	3.54109	47
2.00	1.00	1.00	76.5000	.92582	8
		2.00	3.7500	.70711	8
		Total	40.1250	37.57637	16
	2.00	1.00	129.8750	.99103	8
		2.00	3.7500	.70711	8
		Total	66.8125	65.13598	16
	3.00	1.00	106.3750	.74402	8
		2.00	4.2500	.70711	8
		Total	55.3125	52.74178	16
	Total	1.00	104.2500	22.32809	24
		2.00	3.9167	.71728	24
		Total	54.0833	53.05149	48
3.00	1.00	1.00	9.7500	.70711	8
		2.00	5.1250	.64087	8
		Total	7.4375	2.47572	16
	2.00	1.00	11.5000	.92582	8
		2.00	6.0000	.75593	8
		Total	8.7500	2.95522	16
	3.00	1.00	11.8750	.83452	8
		2.00	6.1250	.64087	8
		Total	9.0000	3.05505	16
	Total	1.00	11.0417	1.23285	24
		2.00	5.7500	.79400	24
		Total	8.3958	2.86386	48
4.00	1.00	1.00	15.2500	.88641	8
		2.00	2.6250	.74402	8
		Total	8.9375	6.56728	16
	2.00	1.00	23.8750	.99103	8
		2.00	2.7500	.70711	8
		Total	13.3125	10.94056	16
	3.00	1.00	18.2500	.70711	8
		2.00	2.8750	.64087	8
		Total	10.5625	7.96634	16
	Total	1.00	19.1250	3.74529	24
		2.00	2.7500	.67566	24
		Total	10.9375	8.69190	48
Total	1.00	1.00	28.2813	28.36653	32
		2.00	4.4688	1.58591	32
		Total	16.3750	23.26350	64
	2.00	1.00	44.9375	50.04639	32
		2.00	4.8438	1.83355	32
		Total	24.8906	40.52595	64
	3.00	1.00	38.3548	40.85466	31
		2.00	5.0313	1.76862	32
		Total	21.4286	33.03357	63
	Total	1.00	37.1789	40.88957	95
		2.00	4.7813	1.73025	96
		Total	20.8953	33.05226	191

EXPERIMENT 1

Serotonin-Striatum

Pruebas de los efectos inter-sujetos

Variable dependiente: HPLC

Origen	Suma de cuadrados tipo III	gl	Media cuadrática	F	Sig.	Eta al cuadrado parcial	Parámetro de no centralidad Parámetro	Potencia observada[b]
Modelo corregido	207460.049[a]	23	9020.002	14229.936	.000	.999	327288.525	1.000
Intersección	83041.542	1	83041.542	131006.158	.000	.999	131006.158	1.000
Grupos	70675.408	3	23558.469	37165.790	.000	.999	111497.371	1.000
Tiempos	2339.859	2	1169.930	1845.678	.000	.957	3691.357	1.000
Serotonin	49334.918	1	49334.918	77830.660	.000	.998	77830.660	1.000
Grupos * Tiempos	3598.673	6	599.779	946.210	.000	.971	5677.259	1.000
Grupos * Serotonin	75069.167	3	25023.056	39476.319	.000	.999	118428.956	1.000
Tiempos * Serotonin	2124.490	2	1062.245	1675.795	.000	.953	3351.591	1.000
Grupos * Tiempos * Serotonin	3757.869	6	626.312	988.068	.000	.973	5928.406	1.000
Error	105.857	167	.634					
Total	290959.000	191						
Total corregida	207565.906	190						

a. R cuadrado = .999 (R cuadrado corregida = .999)

b. Calculado con alfa = .05

Estadísticos descriptivos

Variable dependiente: HPLC

Grupos	Tiempos	Serotonin	Media	Desviación típica	N
1.00	1.00	1.00	211.3750	1.76777	8
		2.00	67.2500	1.03510	8
		Total	139.3125	74.43899	16
	2.00	1.00	214.6250	2.77424	8
		2.00	72.5000	1.92725	8
		Total	143.5625	73.42930	16
	3.00	1.00	213.7143	1.49603	7
		2.00	31.3750	1.18773	8
		Total	116.4667	94.16844	15
	Total	1.00	213.2174	2.46718	23
		2.00	57.0417	18.71869	24
		Total	133.4681	80.03473	47
2.00	1.00	1.00	535.6250	6.23212	8
		2.00	28.2500	.70711	8
		Total	281.9375	262.04236	16
	2.00	1.00	854.0000	1.06904	8
		2.00	36.2500	.70711	8
		Total	445.1250	422.28519	16
	3.00	1.00	211.0000	.92582	8
		2.00	68.1250	.64087	8
		Total	139.5625	73.78434	16
	Total	1.00	533.5417	268.17693	24
		2.00	44.2083	17.60677	24
		Total	288.8750	310.61467	48
3.00	1.00	1.00	209.8750	1.12599	8
		2.00	65.2500	.70711	8
		Total	137.5625	74.68955	16
	2.00	1.00	211.6250	1.40789	8
		2.00	76.1250	.83452	8
		Total	143.8750	69.98083	16
	3.00	1.00	210.8750	.99103	8
		2.00	68.1250	.83452	8
		Total	139.5000	73.72110	16
	Total	1.00	210.7917	1.35066	24
		2.00	69.8333	4.76095	24
		Total	140.3125	71.30908	48
4.00	1.00	1.00	349.3750	6.23212	8
		2.00	22.7500	1.66905	8
		Total	186.0625	168.72600	16
	2.00	1.00	368.5000	2.20389	8
		2.00	32.3750	1.06066	8
		Total	200.4375	173.58225	16
	3.00	1.00	372.2500	.88641	8
		2.00	28.3750	1.59799	8
		Total	200.3125	177.58067	16
	Total	1.00	363.3750	10.87403	24
		2.00	27.8333	4.26988	24
		Total	195.6042	169.74308	48
Total	1.00	1.00	326.5625	135.53572	32
		2.00	45.8750	20.83383	32
		Total	186.2188	171.06069	64
	2.00	1.00	412.1875	267.06378	32
		2.00	54.3125	20.44258	32
		Total	233.2500	260.43803	64
	3.00	1.00	253.1935	71.39254	31
		2.00	49.0000	19.49028	32
		Total	149.4762	115.08859	63
	Total	1.00	331.4632	188.29000	95
		2.00	49.7292	20.35241	96
		Total	189.8586	194.15066	191

EXPERIMENT 1

Serotonin-PFC

Pruebas de los efectos inter-sujetos

Variable dependiente: HPLC

Origen	Suma de cuadrados tipo III	gl	Media cuadrática	F	Sig.	Eta al cuadrado parcial	Parámetro de no centralidad Parámetro	Potencia observada[b]
Modelo corregido	7161123,38[a]	23	311353.190	62811.982	.000	1.000	1444675.580	1.000
Intersección	6888945.855	1	6888945.855	1389766.845	.000	1.000	1389766.845	1.000
Grupos	730358.141	3	243452.714	49113.829	.000	.999	147341.487	1.000
Tiempos	218826.173	2	109413.087	22072.852	.000	.996	44145.703	1.000
Serotonin	3754504.121	1	3754504.121	757428.706	.000	1.000	757428.706	1.000
Grupos * Tiempos	533186.748	6	88864.458	17927.398	.000	.998	107564.391	1.000
Grupos * Serotonin	974432.659	3	324810.886	65526.920	.000	.999	196580.759	1.000
Tiempos * Serotonin	190292.512	2	95146.256	19194.680	.000	.996	38389.360	1.000
Grupos * Tiempos * Serotonin	722800.725	6	120466.788	24302.811	.000	.999	145816.864	1.000
Error	827.804	167	4.957					
Total	14046795,00	191						
Total corregida	7161951.183	190						

a. R cuadrado = 1.000 (R cuadrado corregida = 1.000)

b. Calculado con alfa = .05

Estadísticos descriptivos

Variable dependiente: HPLC

Grupos	Tiempos	Serotonin	Media	Desviación típica	N
1.00	1.00	1.00	161.3750	1.06066	8
		2.00	60.8750	.83452	8
		Total	111.1250	51.90617	16
	2.00	1.00	84.5000	1.06904	8
		2.00	47.2500	.70711	8
		Total	65.8750	19.25574	16
	3.00	1.00	134.8750	.83452	8
		2.00	55.0000	.75593	8
		Total	94.9375	41.25444	16
	Total	1.00	126.9167	32.58423	24
		2.00	54.3750	5.74693	24
		Total	90.6458	43.35086	48
2.00	1.00	1.00	731.2500	1.03510	8
		2.00	31.8750	.99103	8
		Total	381.5625	361.15702	16
	2.00	1.00	1004.6250	1.06066	8
		2.00	24.5000	.53452	8
		Total	514.5625	506.13502	16
	3.00	1.00	915.1250	.83452	8
		2.00	26.8750	.83452	8
		Total	471.0000	458.69104	16
	Total	1.00	883.6667	116.25148	24
		2.00	27.7500	3.23365	24
		Total	455.7083	440.07233	48
3.00	1.00	1.00	169.7500	1.03510	8
		2.00	55.5000	1.30931	8
		Total	112.6250	59.00946	16
	2.00	1.00	91.3750	.74402	8
		2.00	46.7500	1.28174	8
		Total	69.0625	23.06648	16
	3.00	1.00	140.8750	.83452	8
		2.00	48.3750	.51755	8
		Total	94.6250	47.77150	16
	Total	1.00	134.0000	33.07041	24
		2.00	50.2083	4.02146	24
		Total	92.1042	48.32921	48
4.00	1.00	1.00	177.6250	2.19984	8
		2.00	26.7500	1.28174	8
		Total	102.1875	77.93093	16
	2.00	1.00	100.3750	1.06066	8
		2.00	18.3750	.51755	8
		Total	59.3750	42.35229	16
	3.00	1.00	150.1250	.64087	8
		2.00	20.3750	.51755	8
		Total	85.2500	67.00497	16
	Total	1.00	142.7083	32.68756	24
		2.00	21.8333	3.73778	24
		Total	82.2708	65.26957	48
Total	1.00	1.00	310.0000	247.17305	32
		2.00	43.7500	14.94722	32
		Total	176.8750	219.49007	64
	2.00	1.00	320.2188	401.50666	32
		2.00	34.2188	13.19515	32
		Total	177.2188	316.51798	64
	3.00	1.00	335.2500	340.19359	32
		2.00	37.6563	14.65432	32
		Total	186.4531	282.03714	64
	Total	1.00	321.8229	332.28669	96
		2.00	38.5417	14.67968	96
		Total	180.1823	274.21244	192

EXPERIMENT 1

Serotonin-VTA

Pruebas de los efectos inter-sujetos

Variable dependiente: HPLC

Origen	Suma de cuadrados tipo III	gl	Media cuadrática	F	Sig.	Eta al cuadrado parcial	Parámetro de no centralidad Parámetro	Potencia observada[b]
Modelo corregido	14361592,0[a]	23	624417.043	622102.673	.000	1.000	14308361,48	1.000
Intersección	6233406.380	1	6233406.380	6210302.576	.000	1.000	6210302.576	1.000
Grupos	4861237.766	3	1620412.589	1614406.612	.000	1.000	4843219.835	1.000
Tiempos	3778.823	2	1889.411	1882.408	.000	.957	3764.817	1.000
Serotonin	3851916.797	1	3851916.797	3837639.863	.000	1.000	3837639.863	1.000
Grupos * Tiempos	190378.094	6	31729.682	31612.078	.000	.999	189672.467	1.000
Grupos * Serotonin	5261932.516	3	1753977.505	1747476.477	.000	1.000	5242429.430	1.000
Tiempos * Serotonin	8036.844	2	4018.422	4003.528	.000	.979	8007.056	1.000
Grupos * Tiempos * Serotonin	184311.156	6	30718.526	30604.669	.000	.999	183628.016	1.000
Error	168.625	168	1.004					
Total	20595167,00	192						
Total corregida	14361760,62	191						

a. R cuadrado = 1.000 (R cuadrado corregida = 1.000)

b. Calculado con alfa = .05

EXPERIMENT 2

IL

1	7
1	6
1	5
1	9
1	8
1	7
1	6
1	8
2	44
2	42
2	45
2	44
2	46
2	44
2	43
2	44
3	18
3	17
3	16
3	20
3	19
3	18
3	19
3	17
4	4.5
4	5.5
4	3.5
4	6.5
4	4.5
4	3.5
4	2.5
4	5.5

ANOVA de un factor

VAR00002

	Suma de cuadrados	gl	Media cuadrática	F	Sig.
Inter-grupos	8712.000	3	2904.000	1767.652	.000
Intra-grupos	46.000	28	1.643		
Total	8758.000	31			

1	4
1	3
1	2
1	6
1	5
1	4
1	5
1	3
2	23
2	22
2	25
2	26
2	24
2	25
2	23
2	24
3	3.125
3	5.125
3	4.125
3	3.125
3	3.125
3	3.125
3	3.125
3	3.125
4	3
4	2
4	4
4	2
4	3
4	4
4	3
4	3

EXPERIMENT 2

VTA

ANOVA de un factor

VAR00002

	Suma de cuadrados	gl	Media cuadrática	F	Sig.
Inter-grupos	2814.344	3	938.115	824.069	.000
Intra-grupos	31.875	28	1.138		
Total	2846.219	31			

1	11
1	8
1	9
1	10
1	8
1	9
1	8
1	9
2	38
2	36
2	39
2	36
2	37
2	36
2	37
2	37
3	9.5
3	10.5
3	11.5
3	7.5
3	8.5
3	9.5
3	10.5
3	8.5
4	8.5
4	6.5
4	7.5
4	6.5
4	4.5
4	7.5
4	4.5
4	6.5

EXPERIMENT 2

AcbSh

ANOVA de un factor

VAR00002

	Suma de cuadrados	gl	Media cuadrática	F	Sig.
Inter-grupos	5686.000	3	1895.333	1263.556	.000
Intra-grupos	42.000	28	1.500		
Total	5728.000	31			

1	11
1	8
1	9
1	10
1	8
1	9
1	8
1	9
2	43
2	42
2	45
2	44
2	43
2	45
2	44
2	46
3	10
3	8
3	9
3	8
3	6
3	9
3	6
3	8
4	7
4	8
4	5
4	4
4	6
4	8
4	5
4	5

EXPERIMENT 2

AcbC

ANOVA de un factor

VAR00002

	Suma de cuadrados	gl	Media cuadrática	F	Sig.
Inter-grupos	19024.000	3	6341.333	3414.564	.000
Intra-grupos	52.000	28	1.857		
Total	19076.000	31			
